# UCP-2 inhibitor enhanced the efficacy of trastuzumab against HER2 positive breast cancer cells

**DOI:** 10.1007/s00280-021-04303-4

**Published:** 2021-06-19

**Authors:** Jun Hua, Zhe Zhang, Lili Zhang, Yan Sun, Yuan Yuan

**Affiliations:** 1 Department of Neurology & Psychology, The Fourth Clinical Medical College of Guangzhou University of Chinese Medicine, 1 Fuhua Road, Shenzhen, 518033 Guangdong China; 2 Department of Chemotherapy, Jiangsu Cancer Hospital, Nanjing, 210009 Jiangsu China; 3 Department of Pathology, Jiangsu Cancer Hospital, Nanjing, 210009 Jiangsu China; 4 Jiangsu Institute of Cancer Research, Nanjing, 210009 Jiangsu China; 5 The Affiliated Cancer Hospital of Nanjing Medical University, Nanjing, 210009 Jiangsu China

**Keywords:** Trastuzumab, Uncoupling protein 2 (UCP-2), Genipin, Human epidermal growth factor receptor 2 (HER2), Breast cancer

## Abstract

**Purpose:**

This study aimed to investigate the possibility of UCP-2 inhibitor in reducing acquired resistance of trastuzumab to improve the outcome of patients receiving trastuzumab therapy by exploring the relationship between UCP-2 expression and HER2 signaling pathway and examining whether UCP-2 expression was modulated by trastuzumab treatment.

**Methods:**

32 women diagnosed with primary HER2-positive breast cancer were recruited in this study. Needle biopsy was obtained from patients before they received at least four cycles neoadjuvant therapy containing trastuzumab in combination with chemotherapy. Surgical tumor biopsy was obtained during surgical procedure after the neoadjuvant therapy. Levels of HER2 phosphorylation and UCP-2 expression were detected by immunohistochemistry (IHC) and compared between tumor needle biopsy tissue and surgical tumor samples of these patients, as well as in BT474 breast cancer cells before and after trastuzumab treatment. HER2-selective phosphorylation/kinase activity inhibitor ONT-380 was used to identify the correlation between HER2 phosphorylation level and UCP-2 expression. UCP-2 inhibitor Genipin was then used to evaluate the apoptosis index in BT474 cells treated with trastuzumab.

**Results:**

UCP-2 expression was significantly elevated in surgical tumor samples from breast cancer patients receiving trastuzumab in a neoadjuvant setting. We further confirmed our findings in HER2-positive BT474 cell line and found that trastuzumab treatment induced phosphorylation of HER2 and the overexpression of UCP-2, and the latter can be reversed by HER2 selective kinase inhibitor ONT-380. Moreover, UCP-2 inhibitor Genipin significantly enhanced the proliferation suppression effects of trastuzumab and markedly promoted apoptosis.

**Conclusion:**

Taken together, our study identified UCP-2 as a novel therapeutic target for HER2 positive breast cancer and UCP-2 inhibitor may have great potential to enhance the response rate and efficacy of trastuzumab therapy.

## Introduction

Human epidermal growth factor receptor 2 (HER2) is a tyrosine kinase receptor that belongs to the transmembrane epidermal growth factor receptor (EGFR) family. HER2 amplification is clinically observed in approximately 20 to 30% of breast cancer patients, and plays a key role in the tumorigenesis, metastasis and chemotherapy resistance in HER2-positive breast cancer [1]. Trastuzumab is a humanized monoclonal antibody specifically targets the extracellular domain IV of HER2, and has been used world-wide for the treatment of HER2-positive breast cancer [2, 3] for the past two decades. The mechanism of tumor growth inhibition by trastuzumab probably involves endocytic degradation of HER2 receptor, inhibition of the downstream survival and proliferative signaling pathways, [4] suppression of angiogenesis [5], and enhancement of antibody-dependent cellular cytotoxicity (ADCC) activity [6].

Although trastuzumab provides clinical benefits to certain subpopulation of patients with HER2-positive breast cancer, initial response rate is only about 30% and over 70% initial responders acquire resistance within 1 year of initial treatment [7] which necessitate the study of resistance mechanism and development of novel combination therapies to enhance the anti-tumor efficacy of trastuzumab or prevent the development of acquired resistance. Growing evidence has indicated that trastuzumab does not abrogate HER2 tyrosine phosphorylation and completely block its kinase activity [8, 9]. Dokmanovic et al. showed that trastuzumab activated HER2 kinase activity in trastuzumab-sensitive HER2 positive breast cancer cell line BT474. They proposed that binding of trastuzumab to HER2 might induce structural changes in HER2 cytoplasmic kinase domain, resulting in the phosphorylation and activation of HER2 kinase activity [10]. However, the precise role of HER2 phosphorylation in trastuzumab efficacy and resistance development remains to be elucidated.

Several studies have shown that mitochondrial UCP*-2* gene is highly expressed in HER2 positive breast cancer patients and cell lines, and elevated UCP-2 expression has been linked to tumor progression, increased resistance to oxidative stress by reducing mitochondrial reactive oxygen species (ROS), and resistance to various anti-tumor drugs [11–13]. A recent report indicated that inhibition of UCP-2 increased oxidative stress and sensitized breast cancer cells to tamoxifen treatment, suggesting UCP-2 as a new therapeutic target in estrogen receptor positive breast cancer [14]. Furthermore, our earlier study provided direct evidences that UCP-2 played an essential role in the development of doxorubicin-resistance in MCF-7 breast cancer cells [15]. On the other hand, whether the expression of UCP-2 plays a role in HER2 mediated tumor growth and UCP-2 modulator can enhance the treatment efficiency of trastuzumab have not been well studied.

In the present study, we evaluated UCP-2 expression in tissue samples from HER2 positive breast cancer patient receiving trastuzumab in a neoadjuvant setting. Furthermore, we investigated the anti-tumor efficacy of combined treatment of UCP-2 inhibitor Genipin with trastuzumab in BT474 cell line.

## Materials and methods

### Tissue samples collection

32 women diagnosed with primary HER2-positive breast cancer without prior treatment were enrolled in our study. Needle biopsies were taken from all the patients before receiving at least four cycles neoadjuvant therapy containing trastuzumab (8 mg/kg for the first time followed by 6 mg/kg q3w) in combination with chemotherapy and surgical biopsy were obtained during surgical operation after neoadjuvant therapy. The needle biopsies and surgical sections were dissected (3 μm thick), formalin-fixed, paraffin embedded and mounted on charged slides (Thermo Scientific) for storage.

Written informed consent was obtained from all patients. Collection of human tissue samples wad conducted in accordance with the International Ethical Guidelines for Biomedical Research Involving Human Subjects. This study was approved by the Ethics Committee of the Nanjing Medical University Affiliated Cancer Hospital.

### Immunohistochemistry

HER2 phosphorylation and UCP-2 expression in tissue samples were examined by immunohistochemistry. Histological sections were deparaffinized and rehydrated, HER2 and UCP-2 antigens were retrieved by immersing in 10 mM citrate buffer, pH 6.0 in a steamer (95℃) for 40 min. The slides were then cooled to room temperature and blocked in 3% hydrogen peroxide and 5% BSA for 15 min and 1 h, respectively. The sections were then incubated with anti-Phospho-HER2 (Tyr1221/1222) (1:300) (Cell Signaling Technology, #2243) or anti-UCP-2 (1:100) (Abcam, #ab97931) at 4 °C overnight, followed by 1 h incubation with corresponding secondary antibody at room temperature labeled with Labeled HER2 tyrosine phosphorylation-Streptavidin–Biotin (LSAB) method (Invitrogen) and visualized by 3,3’-diaminobenzidine (ImmPACT DAB, Vector Laboratories). The pictures were captured by semi-automated system (Stereo Investigator, MicroBright Field. Inc., Williston, VT, U.S.A.).

### Cell culture and treatments

BT474 cells were obtained from the American Type Culture Collection (ATCC) and maintained as recommended by the manufacturer. All cell cultures were routinely checked for the absence of mycoplasma contamination. Exponentially growing BT474 cells were trypsinized and seeded in 96-cell or 6-well plates at a density of 10, 000 cells/well for proliferation assays, flow cytometry or western blot HER2 tyrosine phosphorylation lotting analysis. Cells were allowed to attach at 37 °C for 24 h before drug treatment. Genipin was obtained from Sigma–Aldrich (CAS: 6902–77-8). Trastuzumab was obtained from the pharmacy department of Jiangsu Cancer Hospital. ONT-380 was obtained from MCE (Cat: HY-16069).

### Transfection of small interfering RNA (siRNA) into BT474 cell line

For HER2 silencing, BT474 cell was transfected with control siRNA (C-siRNA, sense: 5’-UUC UCC GAA CGU GUC ACG UTT-3’; antisense: 5’-ACG UGA CAC GUU CGG AGA ATT-3’, GenePharma, China) nontargeting siRNA or a set of four human HER2-specific siRNAs (primer 1 sense: 5’-GCA GUU ACC AGU GCC AAU ATT-3’; antisense: 5’-UAU UGG CAC UGG UAA CUG CTT-3’; primer 2 sense: 5’-GGU GUA UGC AGA UUG CCA ATT-3’; antisense: 5’-UUG GCA AUC UGC AUA CAC CTT-3’; primer 3 sense: 5’-GCU CUU UGA GGA CAA CUA UTT-3’; antisense: 5’-AUA GUU GUC CUC AAA GAG CTT-3’, GenePharma, China) using Lipofectamine 2000 (Invitrogen), and evaluated 48 h later. For UCP-2 silencing, BT474 cell was transfected with control-siRNA nontargeting siRNA or a set of four human UCP-2-specific siRNAs (primer 1 sense: 5’-GGU AAA GGU CCG AUU CCA ATT-3’; antisense: 5’-UUG GAA UCG GAC CUU UAC CTT-3’; primer 2 sense: 5’-GCA GCG CCA AAU GAG CUU UTT-3’; antisense: 5’-AAA GCU CAU UUG GCG CUG CTT-3’; primer 3 sense: 5’-CCC UUA CCA UGC UCC AGA ATT-3’; antisense: 5’-UUC UGG AGC AUG GUA AGG GTT-3’(GenePharma, China) using Lipofectamine 2000 (Invitrogen), and evaluated 24 h later.

Transient transfection of siRNA was performed according to the manufacturer’s protocol (Invitrogen & GenePharma). Briefly, siRNAs were dissolved in siRNA buffer to prepare a 10 μM stock solution. BT474 grown in 6-well plates were transfected with siRNA in transfection medium (Gibco/Invitrogen) containing liposomal transfection reagent (Lipofectamine RNAimax, Invitrogen). For each transfection, 100 μL of transfection medium containing 4 μL of siRNA stock solution was gently mixed with 100 μL of transfection medium containing 4 μL of transfection reagents. After a 30-min incubation at room temperature, siRNA-lipid complexes were added to the cells in 1.0 mL of transfection medium, and then cells were incubated with this mixture for 6 h at 37 °C. The transfection medium was then replaced with normal medium, and cells were cultured for 24 or 48 h.

### Cell proliferation assay

After seeded in 96-well plate for 24 h, cells were treated with DMSO or Genipin (3 and 10 μM) in the presence of a threefold series dilution from the 0.2 mg/ml of trastuzumab for 72 h, and cell viability was assessed using the CellTiter Aqueous assay with MTS tetrazolium (Promega Corporation), in which viable cells convert MTS tetrazolium into a formazan-colored product at an absorbance of 490 nm. All experiments were done in triplicates. IC50 values for cell lines were calculated with Prism 5 (GraphPad) using the sigmoidal dose–response (variable slope) curve equation. Cells treated with DMSO were used as controls.

### Flow cytometry

BT474 cells (1 × 10^6^) were seeded in 6-well plates for 24 h before treating with DMSO or Genipin (10 μM) with or without trastuzumab (0.1 and 1 μg/mL) for 72 h. The cells were lyzed and double stained by Annexin V-fluorescein isothiocyanate (FITC) and propidium iodide staining kit (BD Pharmingen, Franklin Lakes, NJ, USA). The samples were analyzed by Guava easycyte flow cytometer (Millipore).

### Western blot analysis

Cells seeded in 6-well plate were treated with trastuzumab (1 μg/mL) with or without ONT-380 (10 μM) for 72 h (Fig. 3), or Genipin (10 μM) with or without trastuzumab (0.1 and 1 μg/mL) for 72 h (Fig. 5). Total protein was prepared by the Total Protein Extraction Kit (Nanjing Keygen, China). Proteins were separated by SDS–PAGE electrophoresis and transferred to PVDF membranes. The blots were blocked by 10% nonfat dry milk in TBS buffer containing 0.1% Tween 20 (for 1 h at room temperature and then incubated with primary antibody as follow: Phospho-HER2 (Tyr1221/1222) (1:1000) (Cell Signaling Technology, #2243), HER2 (1:1000) (Cell Signaling Technology, #2242), Cleaved Caspase-8 (Asp391) (1:1000) (Cell Signaling Technology, #9496), Caspase-3 (1:1000) (Cell Signaling Technology, #9662), PARP(1:1000) (Cell Signaling Technology, #9532), or GAPDH (1:1000) (Cell Signaling Technology, #5174) overnight at 4 °C, followed by corresponding secondary antibody at room temperature for 1 h and visualized by HRP reagents using Omega16IC (Ultra-Lum, Claremont, CA, U.S.A.).

### Quantitative reverse transcription PCR (qRT-PCR)

Cells seeded in 24-well (1 × 10^5^) plate were treated with trastuzumab (1 μg/mL) with or without ONT-380 (10 μM) for 72 h (Fig. 3). Total RNA was prepared by the Trizol reagent (sigma) according to manufacturer’s instructions. First strand of cDNA was synthesized by ChamQ SYBR qPCR Master Mix reverse transcriptase PCR kit (Vazyme, #Q341-02). Quantitative PCR was performed using ABI StepOnePlus qPCR instrument with the following primers:

UCP-2 forward: 5'-CCGGTTACAGATCCAAGGAGAA-3'; reverse: 5'-TCAGAATGGTGCCCATCACA-3';

GAPDH forward: 5'-CCACTCCTCCACCTTTGAC-3'; reverse: 5'-ACCCTGTTGCTGTAGCCA-3'.

### Statistical analysis

**Fig. 1:**
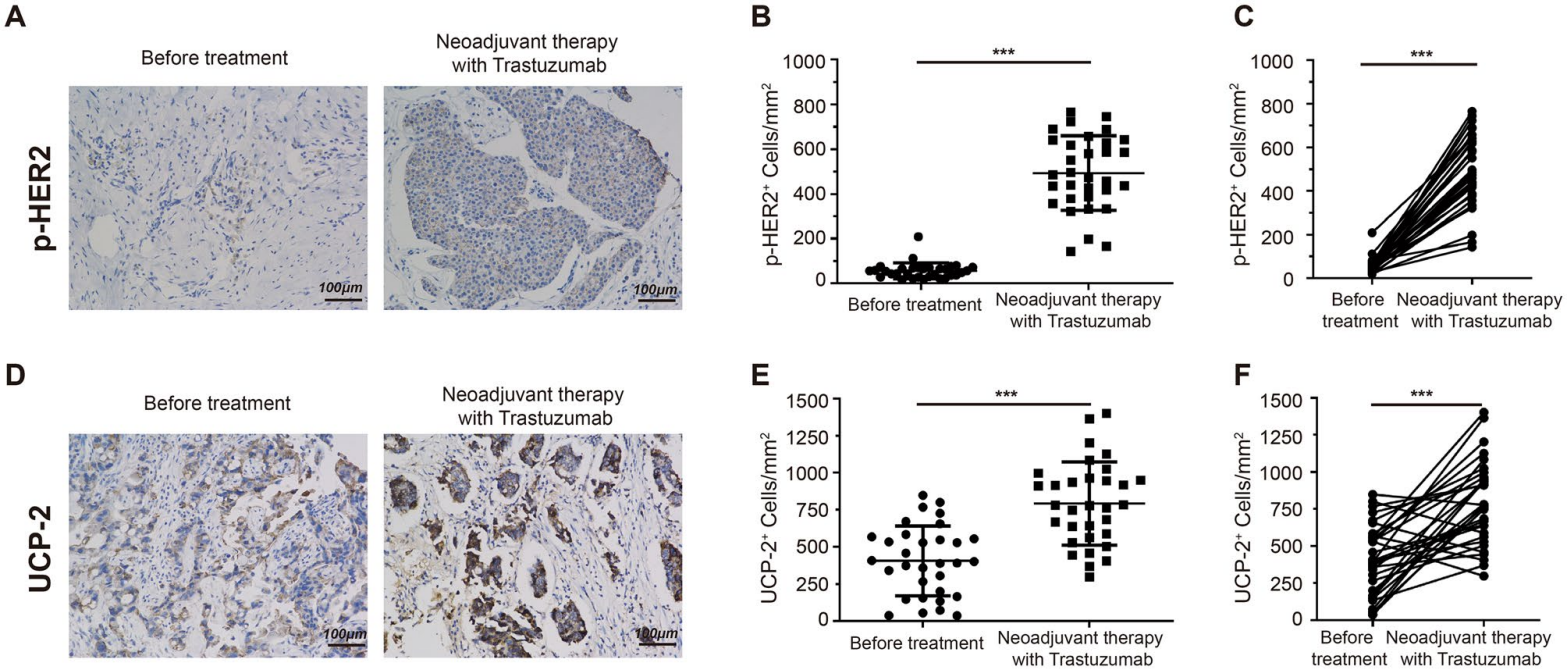
HER2 phosphorylation and UCP-2 expression levels in breast cancer tissue samples measured by IHC. **a–c** HER2 phosphorylation level at Y1221/Y1222 and **d**–**f** UCP-2 expression was determined by IHC (Brown). Representative images from one patient were presented. Scale bar: 100 μm. **b**, **e** Quantification of HER2 phosphorylation and UCP-2 expression before and after receiving trastuzumab. Pared-samples *T* test, ***p < *0.001* vs. before treatment group (*n* = 32). **c**, **f** Graphic displaying individual patient’s level of HER2 phosphorylation or UCP-2 expression pre- and post-treatment

Values were presented as mean ± standard deviation (SD) and analyzed using paired-samples T test at significance level of 0.05 in Fig. 1, and mean ± standard error of the mean (S.E.M.) and analyzed using student’s *t* test at significance level of 0.05 in other results.

## Results

### Trastuzumab increased HER2 phosphorylation and UCP-2 expression in tissue samples from breast cancer patients received neoadjuvant therapy including trastuzumab

To evaluate whether UCP-2 expression was modulated by trastuzumab treatment and whether such change was downstream of HER2 signaling pathway, we first examined HER2 phosphorylation level and UCP-2 expression in tissue samples from HER2-positive breast cancer patients before and after receiving trastuzumab in a neoadjuvant therapy setting by immunohistochemistry (IHC). As shown in Fig. 1, both HER2 phosphorylation level as well as the expression of UCP-2 were significantly upregulated after trastuzumab treatment compared to treatment naive sample from the same patients. Densitometric quantification showed 8.77-fold increase in phospho-HER2 (p-HER2) (*p* < 0.001, *t* = 15.33) and 1.95-fold increase in UCP-2 (*p* < 0.001, *t* = 6.107) expressed cells.

### Trastuzumab increased UCP-2 expression in BT474 breast cancer cells and the increase was abolished by HER2 kinase inhibitor ONT-380

**Fig. 2:**
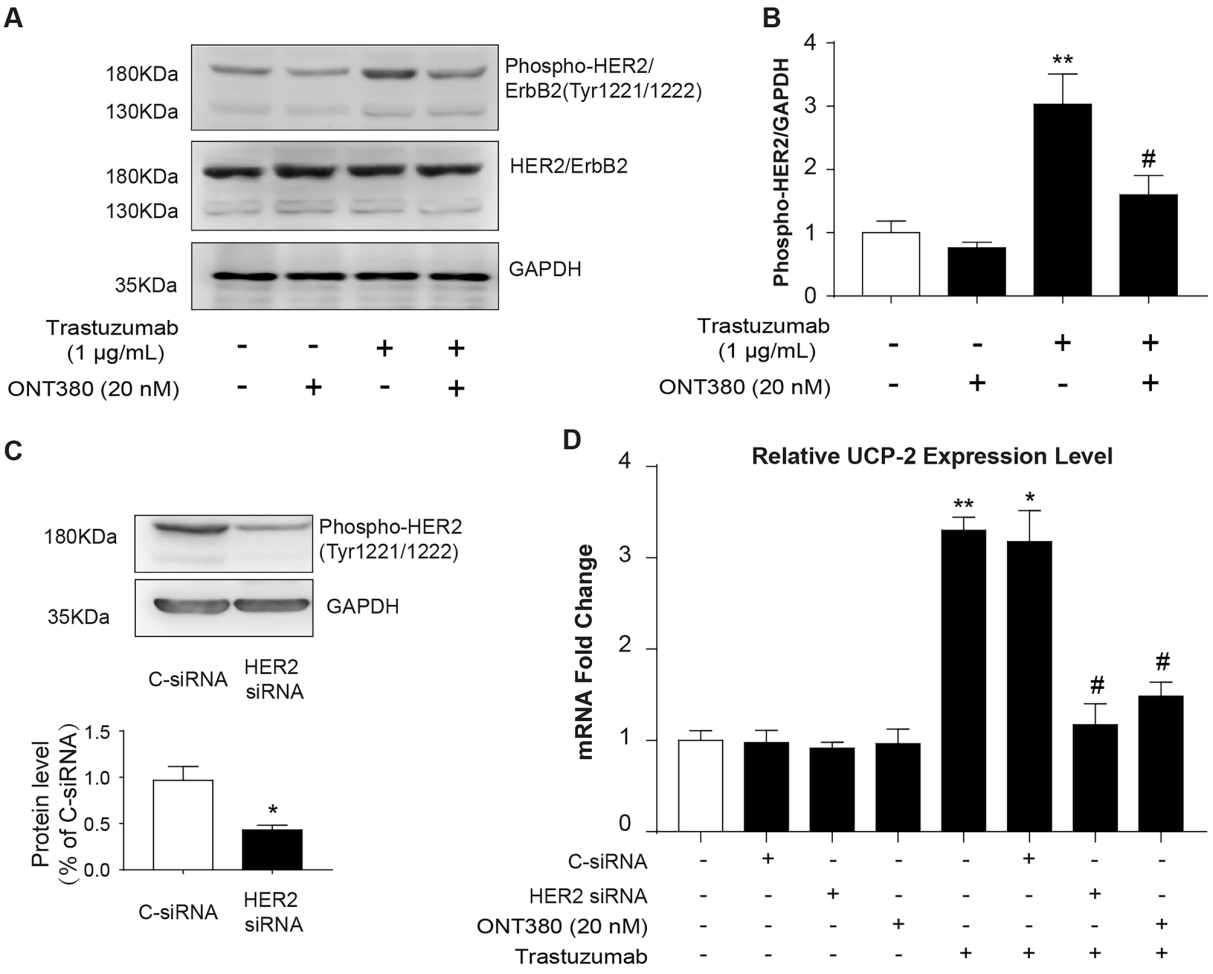
UCP-2 expression was upregulated in trastuzumab-treated BT474 cells in a HER2 phosphorylation dependent manner. **a** Representative western immunoblots of HER2 and phospho-HER2 (p-HER2) in BT474 cells after trastuzumab treatment for 48 h with or without HER2 phosphorylation inhibitor ONT-380 (100 nM). **b** Quantification of p-HER2 levels with GAPDH served as a loading control. **p < *0.01* compared to no treatment group; ^*#*^*p* < *0.05* vs. Trastuzumab treatment group; Data were expressed as mean ± S.E.M, *n* = 3. **c** HER2 protein level in BT474 cell line transfected with control siRNA (C-siRNA) or HER2 siRNA for 48 h. *p < *0.05* compared to C-siRNA treatment group; **d** BT474 cells were transfected with C-siRNA or HER2 siRNA or treated with trastuzumab and with or without ONT-380 for 48 h, and the expression of UCP-2 was examined by qRT-PCR. *p < 0.05, **p < *0.01* vs. No treatment group; ^*#*^*p* < *0.05* vs. Trastuzumab treatment group; Data were expressed as mean ± S.E.M, *n* = 4

To further investigate whether increased expression of UCP-2 was a result of elevated HER2 phosphorylation, we treated HER2 positive breast cancer cell line BT474 with trastuzumab in vitro and examined the expression of p-HER2 and UCP-2 using western blot and qRT-PCR. As shown in Fig. 2a, b, the level of p-HER2 was markedly increased after trastuzumab (1 μg/mL) treatment for 48 h, and the increase was abolished by HER2-selective phosphorylation/kinase activity inhibitor ONT-380 (Irbinitinib, 100 nM).

We then used HER2 siRNA to investigate the specific relationship between HER2 phosphorylation and UCP-2 expression (Fig. 2c, d). Consistent with our findings in patient tissue samples, we found that the expression level of UCP-2 in BT474 cells significantly increased by approximately threefold in trastuzumab-treated group compared to no treatment group at mRNA level via qRT-PCR (Fig. 2d). Moreover, ONT-380 (100 nM) was able to reverse the trastuzumab-induced upregulation of UCP-2. These findings are consistent with our hypothesis that increase of UCP-2 depends on trastuzumab mediated HER2 phosphorylation.

### UCP-2 inhibitor Genipin synergistically enhanced trastuzumab-induced inhibition of BT474 cell growth

**Fig. 3:**
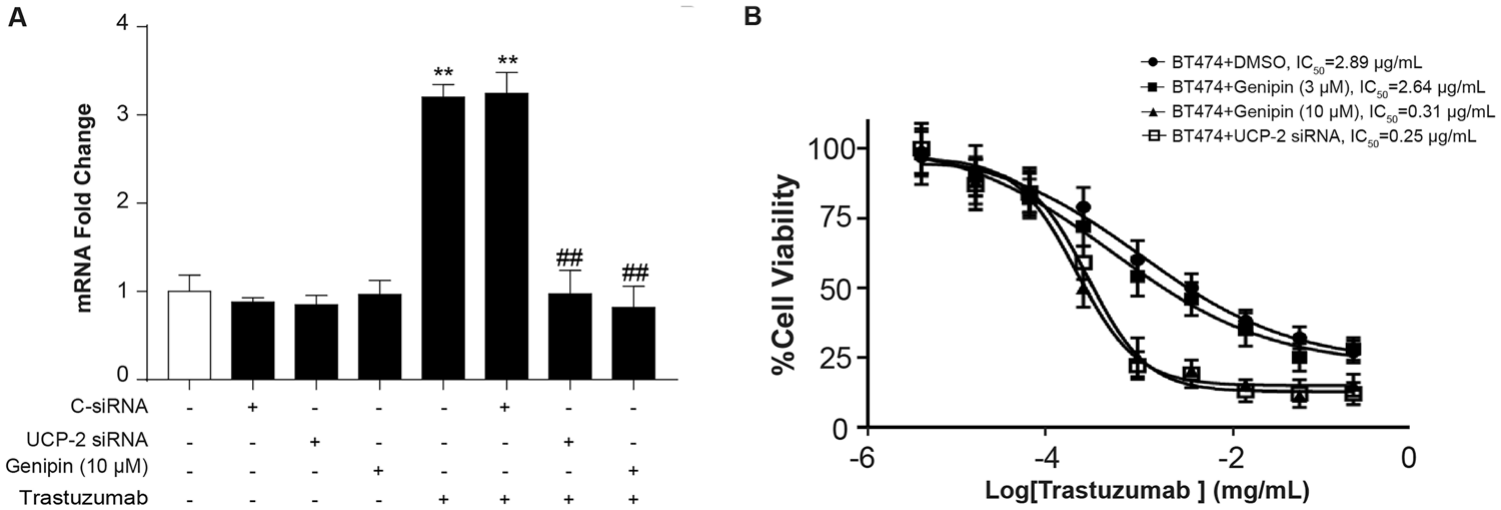
Genipin enhanced trastuzumab sensitivity of BT474 cells. **a** BT474 cells were transfected with control siRNA (C-siRNA), UCP-2 siRNA or treated with trastuzumab and with or without ONT-380 for 24 h, and the expression of UCP-2 was examined by qRT-PCR.*p < 0.05, **p < *0.01* vs. No treatment group; ^*##*^*p* < *0.01* vs. Trastuzumab treatment group; Data were expressed as mean ± S.E.M, *n* = 4. **b** BT474 cells were treated with various concentrations of either trastuzumab alone or in combinations with Genipin (3, 10 μM). Cell viability was evaluated by MTS assays. Data represent mean ± S.E.M. All assays were performed in triplicates

Considering the critical roles of UCP-2 in cancer progression and the potential correlation between HER2 signaling and UCP-2 expression, we proposed that suppression of UCP-2 might be a feasible way to increase initiate response rate and/or enhance efficacy in trastuzumab in breast cancer therapy. We treated BT474 cells with control siRNA (C-siRNA), UCP-2 siRNA, trastuzumab, and with or without ONT-380 for 48 h, respectively, and the expression of UCP-2 was examined by qRT-PCR. As shown in Fig. 3a, Genipin displayed a similar ability to inhibit UCP-2 expression with UCP-2 siRNA.

We then investigated the effects of combined treatment of UCP-2 inhibitor Genipin and trastuzumab on cell viability in BT474 cells (Fig. 3). As expected, the proliferation of BT474 cells was significantly decreased after being treated with varied concentrations of trastuzumab with estimated IC_50_ of 2.89 μg/mL. In the presence of 10 μM Genipin, the IC_50_ shifted significantly by about 10 folds to 0.31 μg/mL. These data strongly supported that Genipin synergistically enhanced trastuzumab-induced inhibition of BT474 cell growth.

### Genipin enhanced the trastuzumab-induced apoptosis of BT474 cells

**Fig. 4:**
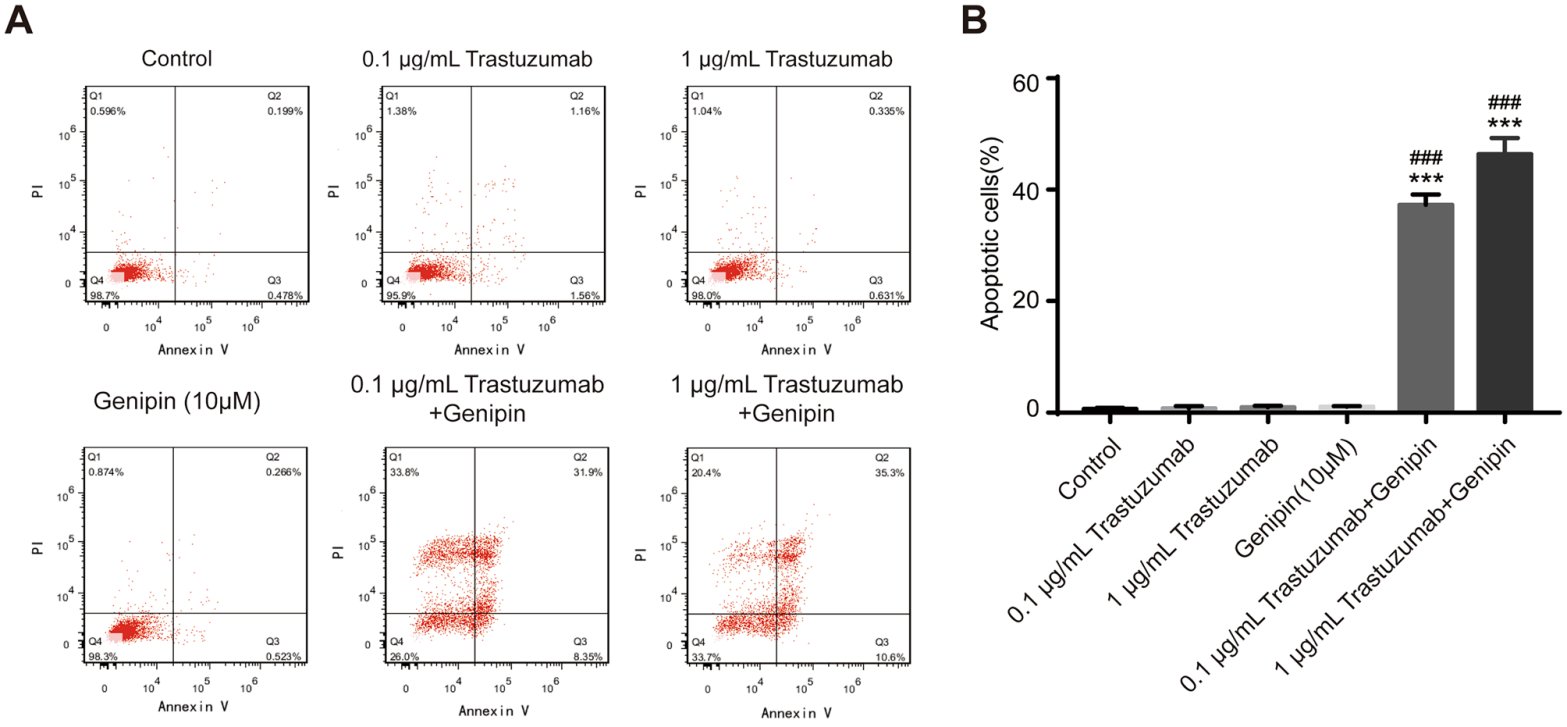
Combination therapy of Genipin and trastuzumab synergistically enhances apoptosis of BT474 cells. **a** Apoptosis analysis in BT474 cells by flow cytometry after Genipin and trastuzumab treatment. Cells were treated with Genipin (10 μM) and/or trastuzumab (0.1 or 1 μg/mL) for 24 h, and stained with PI and Annexin V-FITC. **b** The positive stained cells were counted using FACScan. ***p < *0.001,* vs. Control group; ^*###*^*p* < *0.001* vs. trastuzumab treatment group, respectively. Data represented mean ± S.E.M of three independent experiments

We next examined whether synergistic effects of Genipin toward trastuzumab resulted in increased cancer cell apoptosis. BT474 cells were treated with Genipin (10 μM), trastuzumab (0.1 or 1 μg/mL) or the combination of both drugs for 24 h, and survival status of BT474 cells were analyzed by flow cytometry using Annexin V-FITC/PI double staining. As shown in Fig. 4a,b, trastuzumab at experimental concentration much lower than the IC_50_ does not significantly induce apoptosis, with early apoptotic cells (Annexin V-positive/PI-negative, Quadrant 1) and late apoptotic/necrotic cells (Annexin V-positive/PI-positive, Quadrant 2) remained around or below 1% of total cells. Genipin alone at 10 μM, which exhibited synergistic effects in the proliferation assay, also did not induce significant cell death.

However, the combination of Genipin and trastuzumab-induced dramatic apoptosis to 37.1% and 46.3% of combined early and late apoptotic cells at 0.1 and 1 μg/mL trastuzumab, respectively. (Fig. 4a, b).

**Fig. 5:**
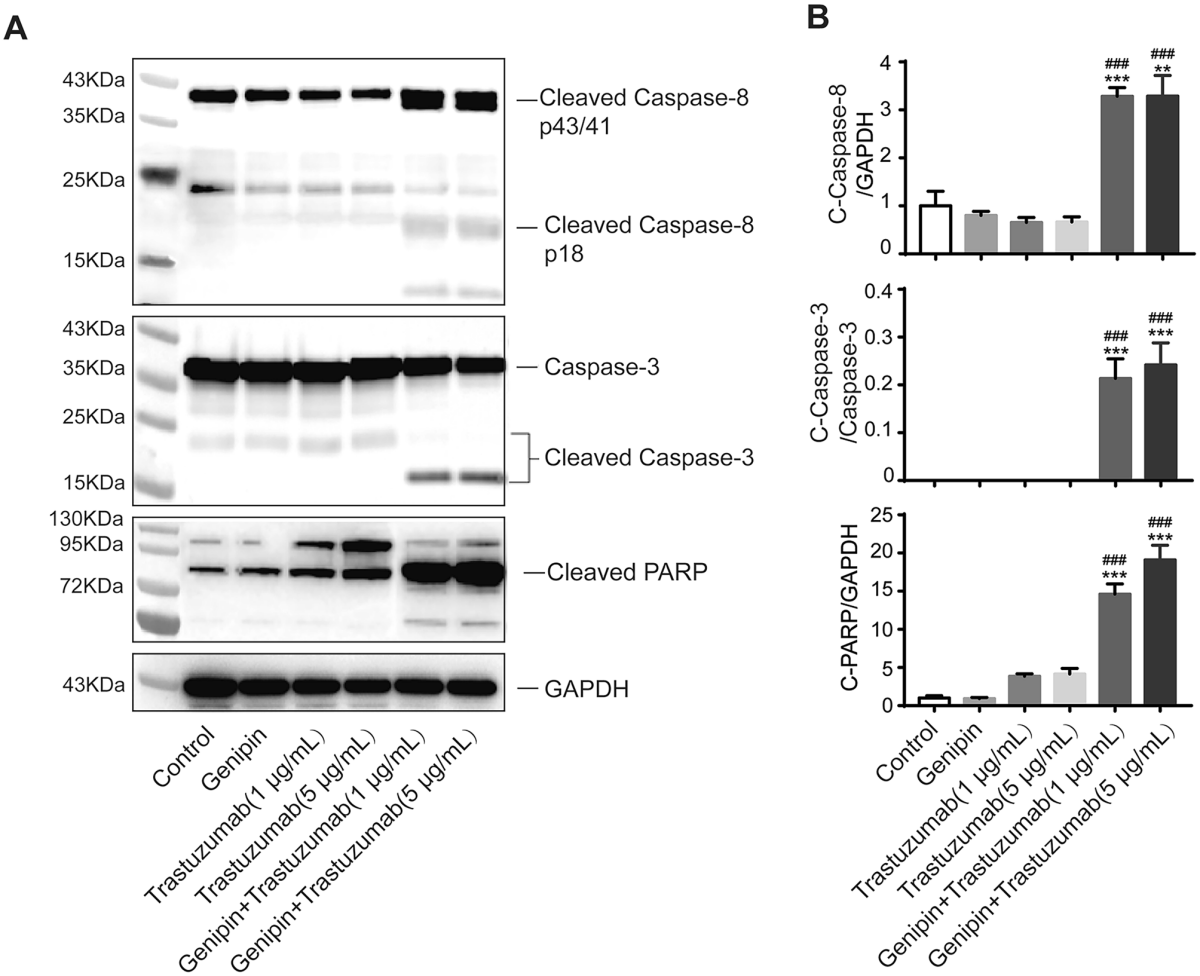
Combination Genipin and trastuzumab increased apoptosis of BT474 cells. **a** Representative western immunoblots of caspase 8, caspase 3 and PARP and their cleaved products in BT474 cells after trastuzumab (1 or 5 μg/mL) treatment for 48 h with or without Genipin (10 μM). **b** Quantification of caspase 8, caspase 3 and PARP and their cleaved products. GAPDH served as a loading control. ***p < *0.001,* vs. Control group; ^*###*^*p* < *0.01* vs. Trastuzumab treatment group; Data represent mean ± S.E.M of three independent experiments

Finally, we examined activated apoptosis-related proteins, including cleaved caspase 8, caspase 3, and PARP using western blot using lysate of cells treated with trastuzumab (1 or 5 μg/mL) in the presence or absence of Genipin (10 μM) for 48 h. As shown in Fig. 5, trastuzumab and Genipin induced moderate level of cleaved caspase 3 and PARP, while the combination of the two drugs dramatically increased the level of cleaved caspase 8, caspase 3 and PARP, indicating significant induction of apoptosis, in consistence with our flow cytometry results. These results indicated that Genipin and trastuzumab synergistically enhanced the apoptosis of BT474 cells.

## Discussion

In the present study, we showed for the first time that trastuzumab treatment induced elevated expression of UCP-2, probably because of HER2 phosphorylation, using samples from HER2-positive breast cancer patient received trastuzumab neoadjuvant therapy. Moreover, we showed that UCP-2 inhibitor Genipin might enhance the tumor suppression effects of trastuzumab and induce apoptosis in BT474 cells, shedding some light on the development of novel combination therapy against trastuzumab resistance HER2 + breast cancer.

The HER2 gene (Her2 or ErbB2) is overexpressed in about 20% ~ 30% of malignant tumors, including breast cancer and gastric cancer [16, 17]. HER2 is a member of the ErbB receptor tyrosine kinase family. Upon homo- or hetero-dimerization with another member of the ErbB receptor, HER2 is able to activate various proliferative and survival signaling pathways, resulting in tumor growth [2]. Trastuzumab is the first generation monoclonal antibody targeting HER2 signaling, and has been used as standard of care treatment for HER2-amplified or highly expressed breast cancer [18, 19]. Trastuzumab binds to the extracellular domain IV of HER2 protein, and blocks HER2 mediated signaling via several possible mechanisms including preventing HER2-receptor dimerization, enhancing receptor endocytosis, and inducing immunological response [2]. Despite the demonstrated tumor suppression effects and improvement of survival by trastuzumab in numerous trials, clinical response rate for trastuzumab monotherapy remains low and both primary and secondary (acquired) resistance have been the major cause of therapy failure. Approximately 30% of early stage and 15% of advanced stage patients respond to trastuzumab monotherapy, respectively. Moreover, among the initial responder [20], approximately 70% of initial responders acquire resistance to the drug within 1 year [18]. Therefore, there is an urgent need to understand the mechanism of resistance and develop novel therapy to prolong the efficacy of or reverse the resistance to trastuzumab.

Although trastuzumab was demonstrated to inhibit HER2 mediated tumor growth, recent studies have shown that trastuzumab does not completely abolish HER2 phosphorylation or its kinase activity [9, 21, 22], and the contribution of HER2 phosphorylation to trastuzumab resistance was not unambiguously elucidated. Dokmanovic et al. showed that trastuzumab-induced HER2 phosphorylation and activated HER2 kinase activity in BT474 cells, while the signaling blockade mechanism was probably a result of receptor endocytosis [10]. Phosphorylated HER2 drove the activation of MAPK/ERK and PI3K/AKT pathways, thereby promoting cellular survival and proliferation, thus might contribute to the acquired resistance to trastuzumab [23, 24]. On the other hand, it was reported that phosphorylation of HER2 correlated negatively with acquisition of resistance to trastuzumab therapy [25], but positively associated with the response rates of trastuzumab and chemotherapy combination [26].

Consistent with Dokmanovic et al. we also found that trastuzumab increased the phosphorylation level of HER2 in patient samples after trastuzumab neoadjuvant therapy compared to trastuzumab-naïve samples. Moreover, we showed that trastuzumab treatment led to overexpression of UCP-2 in both patient samples and BT474 cells, and the elevation of UCP-2 expression could be reversed by HER2 kinase activity inhibition and was thus a downstream event of the latter. UCP-2 belongs to the mitochondrial uncoupling protein family, which separates oxidative phosphorylation from ATP synthesis thus releases energy in the form of heat. In normal cells, mitochondrial uncoupling is employed by the cells to diminish ROS production. Overexpression of UCP-2 has been reported in various types of cancers, including HER2-amplified cancers, and is likely to contribute to oxidative stress response, thus related to drug resistance [27, 28]. Here we report for the first time that UCP-2 overexpression was induced by HER2 phosphorylation in HER2-positive breast cancer, and this could be reversed by HER2 kinase inhibitor ONT-380, indicating potential signaling regulation of UCP-2 expression by HER2 phosphorylation. Nirav Patel etc. [31] had reported that the sustained ligand activation of ERBB2/ERBB3 as well as constitutive ERBB2 signaling in BT474 cells could increase the steady state levels of UCP-2 mRNA and protein. This suggested that HER2 phosphorylation cascades were likely involved in the regulation of UCP-2 at the transcription levels, this could be studied in more detailed in the future.

To further investigate whether the inhibition of UCP-2 may enhance the efficacy of trastuzumab, we employed Genipin, the major active component of *Gardeniae fructus* that was previously reported to inhibit mitochondrial uncoupling by inhibiting UCP-2 in isolated pancreatic islets. We found that Genipin significantly enhanced trastuzumab-induced BT474 cell growth inhibition and apoptosis, as evidenced by both an increased population of Annexin V-positive cells in flow cytometry as well the activation of mitochondria-initiated caspase cascades including expression of cleaved caspase 8, caspase 3 and PARP in western blotting analysis. Our data suggested that the combination therapy of Genipin and trastuzumab might exhibit synergistically higher anti-tumor efficacy compared to trastuzumab single therapy. It is worth noting that we and others previously reported that UCP-2 overexpression was also related to doxorubicin-resistance and UCP-2 inhibition could sensitize the cells to chemotherapy treatment via increased mitochondrial membrane potential (ΔΨm), ROS production, apoptosis, and autophagy [15, 29, 30]. Further studies will be needed to confirm the sensitization effects of Genipin with trastuzumab and chemotherapy in vivo and to understand the mechanisms of UCP-2 in therapeutic resistance.

In conclusion, we have shown that trastuzumab could induce UCP-2 overexpression by increasing HER2 phosphorylation. Importantly, the combined therapy of trastuzumab and UCP-2 inhibitor Genipin significantly suppressed tumor cell growth and promoted apoptosis in vitro, indicating that UCP-2 inhibition might be a novel therapeutic target to enhance anti-tumor efficacy of trastuzumab.

## Data Availability

All data generated or analyzed during this study are included in this published article.
